# Electroconvulsive therapy in children and adolescents

**DOI:** 10.1192/j.eurpsy.2022.1130

**Published:** 2022-09-01

**Authors:** M. Esperesate Pajares, C. Pastor Jordá, M. Taracena Cuerda, R. Puente García, A.M. Jiménez Bidón

**Affiliations:** Hospital 12 de Octubre, Psychiatry, Madrid, Spain

**Keywords:** ECT, Adolescents, Electroconvulsive therapy, Children

## Abstract

**Introduction:**

Despite its good results and tolerability in adults, electroconvulsive therapy (ECT) is barely administered in children and adolescents, with scarce evidence in these patients.

**Objectives:**

We aim to summarize the data available to give a clearer view of how children and adolescents might benefit from ECT.

**Methods:**

We’ve done a bibliographic review in PubMed and Cochrane Library searching for articles that include the terms “electroconvulsive therapy” and “adolescents” and/or “children” and their variations.

**Results:**

Current evidence supports the use of ECT in various indications as mood disorders, schizophrenia spectrum disorders, catatonia, neuroleptic malignant syndrome and self-injurious behaviours associated with autism, Tourette’s syndrome or intellectual disability. The efficacy and safety it’s comparable to adults and there are no absolute contraindications. Side-effect profile it’s also similar to the general population, reporting as the most frequent adverse effects headache, generalized body aching, and nausea or vomiting.

**Conclusions:**

ECT is an effective and safe treatment for severe mental disorders in children and adolescents.

**Disclosure:**

No significant relationships.

